# Plasma Membrane-Localized Calcium Pumps and Copines Coordinately Regulate Pollen Germination and Fertility in Arabidopsis

**DOI:** 10.3390/ijms19061774

**Published:** 2018-06-15

**Authors:** Yun Li, Jinping Guo, Ziyuan Yang, Dong-Lei Yang

**Affiliations:** 1 State Key Laboratory of Crop Genetics and Germplasm Enhancement, Nanjing Agricultural University, Nanjing 210095, China; 2015101147@njau.edu.cn (Y.L.); 2016101151@njau.edu.cn (Z.Y.); 2 Department of Anatomy, The Second Military Medical University, Shanghai 200433, China; guojinping81@126.com

**Keywords:** autoinhibited Ca^2+^ ATPase (ACA), calcium, calcium pump, copine, fertility, pollen germination

## Abstract

Calcium plays an important role in plant growth, development, and response to environmental stimuli. Copines are conserved plasma membrane-localized calcium-binding proteins which regulate plant immune responses and development. In this study, we found that copine proteins BON2 and BON3, the paralogs of BON1, physically interact with calcium pumps ACA8 and ACA10 in Arabidopsis. Notably, ACA9, the closest homologue of ACA8 and ACA10 functioning in pollen tube growth, interacts with all three copines. This is consistent with the protein–protein interactions between the two protein families, the *aca8*, *aca10*, *aca8/aca10*, *bon1/2/3* mutants as well as *aca9* mutant exhibited defects on pollen germination and seed production. Taken together, plasma membrane-localized interacting calcium pumps and copines coordinately control pollen tube growth, likely through manipulating calcium efflux.

## 1. Introduction

Ca^2+^ is essential for eukaryotic cells as an important secondary messenger or a structural component of enzymes and macromolecular complexes [1,2,3]. Calcium carries specific information response to environmental and endogenous cues through the amplitude, frequency, and duration of calcium spikes [2,4]. This Ca^2+^ signature is shaped by the coordinated actions of membrane transport proteins in Ca^2+^ influx and efflux systems including channels, pumps, and exchangers [3,5]. Several ion channels, such as Ca^2+^ permeable cyclic nucleotide-gated channels and voltage-gated channels, locate at the plasma membrane (PM), endoplasmic reticulum (ER), vacuole, or mitochondria and control calcium influx [6,7,8]. Calcium efflux can be mediated by Ca^2+^ ATPase pumps and Ca^2+^/H^+^ exchangers [5]. In Arabidopsis thaliana, there are 14 putative Ca^2+^ ATPase pumps that can be divided into two distinct subfamilies, the autoinhibited calcium ATPases (ACAs) and the endoplasmic reticulum-type Ca^2+^ ATPases (ECAs) [9,10]. The autoinhibitory domain at the N-terminal ACA represses its ion pump activity. When calmodulin binding to the autoinhibitory domain, the calcium pump activity was released [11,12,13]. Among the 10 ACAs in Arabidopsis, ACA8, ACA9, ACA10 belong to one clade and localize on the plasma membrane [14,15,16].

ACA genes are involved in multiple biological processes, including plant growth, development, and immunity. ACA7, ACA9, and ACA13, which are located at the plasma membrane, have been shown to be important for pollen development [15,17,18]. ACA9 specifically expresses in pollen and plays a key role in the regulation of pollen tube growth and fertilization [15]. The loss of function mutant of ACA7 produces significant levels of dead pollen grains in mature flowers, suggesting its important role in pollen development [17]. The mutation in *ACA13* causes defects both in pollination and seed production [18]. ACA10 and ACA8 play major roles in vegetative growth and immunity [9,19]. In addition, ACA8 interacts with FLS2 and functions in disease resistance response [10]. ACA13 and ACA12 are also involved in disease resistance and growth, especially when the function of ACA10 and/or ACA8 is compromised [9]. In addition, the mutation of ACA13 and ACA10, together with a reduced function of ACA8, result in death at bolting time, revealing the essential roles of their collective function in plant growth [9]. ACA4 and ACA11 reside at vacuole, the double mutant of *aca4aca11* displays a high frequency of hypersensitive response-like lesions associated with the activation of the salicylic acid pathway [6,20]. 

Copines are evolutionarily conserved proteins in protozoa, plants, nematodes, and mammals [21]. The *BON1/CPN1* in Arabidopsis was the first reported copine gene which acts as a repressor of plant autoimmunity [22,23]. There are two additional copine genes in the Arabidopsis genome, designated as *BON2* and *BON3* [22]. The three genes have overlapped functions which are essential for the viability of plants [22]. The loss of function of BON1, combined with BON2 or BON3, leads to extensive cell death, resembling the hypersensitive response seen in defense responses; however, no abnormal growth or development phenotypes are found in single *bon2* or *bon3* mutants under normal conditions [22].

Recently, we found that the von Willebrand factor type A (VWA)domain of BON1 physically interacts with the autoinhibited domain (ID) of ACA10 and ACA8, suggesting another regulatory mechanism of calcium pump activity except calmodulin binding [19]. Consistent with physical interaction between BON1 and ACA8/10, the mutation in BON1, ACA8, and ACA10 compromised the production of calcium signature in guard cell upon external calcium treatment [19]. Consequently, the mutants exhibited defects on stomatal closure and inhibition of constitutive defense response. This uncovered a critical role for ACA8 and ACA10 in calcium signature generation as well as their regulation by an evolutionarily conserved BON1 protein in Arabidopsis [19]. 

In this study, we also found that BON2 and BON3 physically interact with ACA8 and ACA10. Interestingly, BON1/2/3 also physically interacts with ACA9, the closest homologue with ACA8/10. Consistent with the protein–protein interaction, the *aca8*, *aca9*, *aca10*, *aca8/aca10*, and *bon1/2/3* mutants exhibited defects on pollen germination and seed production. These results demonstrate the coordination between ACA pumps and copines in pollen growth, likely via calcium signaling.

## 2. Results and Discussion

Given that BON1 genetically and physically interacts with ACA8/10 [19], it is possible that the other two copines interact with plasma membrane-localized ACA pumps. To test the hypothesis, we performed split-LUC assay, a widely used method to test protein–protein interaction in tobacco leaves [24]. The coding regions of BON2 and BON3 without stop codon were respectively ligated into the pCAMBIA-NLuc vector [24], and the amino-terminal part of ACA8 (1-106aa, Supplementary Materials, Figure S1A) was ligated into the pCAMBIA-CLuc vector [24]. When the fused protein of BON2 and BON3 with the amino-terminus of firefly luciferase coexpressed with the fused protein of segment I of ACA8 (1–106aa, Supplementary Materials Figure S1A) with carboxyl-terminus of luciferase, it showed strong luminescence signal, suggesting the protein–protein interaction between ACA8 and BON2 and BON3 (Supplementary Materials Figure S2A). In addition, the split-LUC assay also demonstrated the protein–protein interaction between N-terminal of ACA10 (1–72aa, Supplementary Materials Figure S1A) and BON2 and BON3 (Supplementary Materials Figure S2B). Given that the segment I of ACA8 and ACA10 contained the autoinhibited domain, the interaction between BON2 and BON3 with ACA8 and ACA10 released the pump’s activity [19].

To confirm the interaction and further locate the domain responsible for the physical interaction, we cloned segment I of ACA8 (1–106aa), ACA10 (1–72aa, Supplementary Materials Figure S1A) into the activation domain vector (AD) and the VWA domain of BON2 (328–586aa), BON3 (326–584aa, Supplementary Materials Figure S1A) into the DNA-binding domain vector (BD) to perform yeast-two-hybrid assay. As we found previously, the BON1-VWA-BD with ACA8-I-AD and BON1-VWA-BD with ACA10-I-AD (Supplementary Materials Figure S1A) yeasts grew well on synthetic defined (SD) medium which lacked four kinds of amino acids (Trp, Leu, His, and Ade) (Supplementary Materials Figure S3); this confirmed that the protein–protein interaction happens on the VWA domain of BON1 (324–578aa) and the autoinhibitory domain of ACA8 and ACA10 (Supplementary Materials Figure S1A). Yeast-two hybrid assay also demonstrated that the VWA domains of BON2-VWA-BD (328–586aa) and BON3-VWA-BD (326–584aa) interact with the autoinhibitory domains of ACA8-I-AD (1–106aa) and ACA10-I-AD (1–72aa, Supplementary Materials Figure S1A; Supplementary Materials Figure S3). The physical interactions between BON1, BON2, BON3 and ACA8, ACA10 raise the possibility that the cooperative role of five proteins in regulating calcium signature, immunity and stomatal movement.

**Figure 1:**
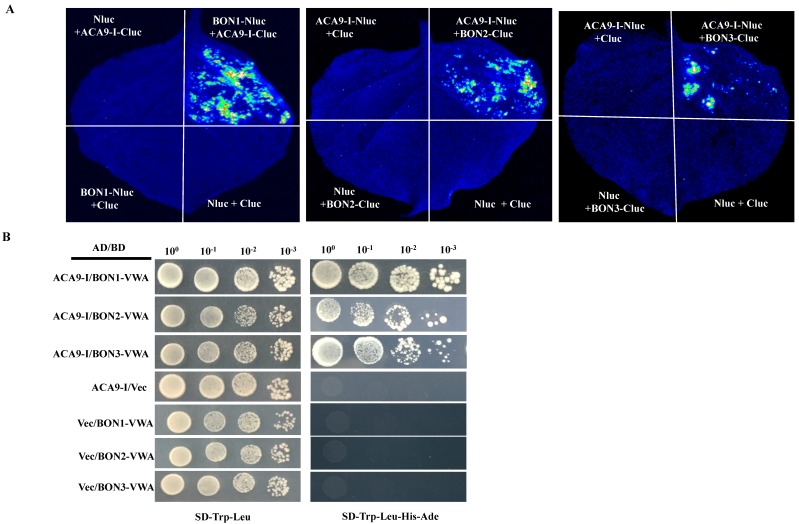
Protein-protein interaction between ACA9 and BON1，BON2，BON3. (**A**). Split-LUC assay of the interaction of BON1, BON2 and BON3 with the first segment of ACA9. The indicated combinations were used as controls. (**B**). Yeast-two-hybrid assay between the first segment of ACA9 and the VWA domain of BON1, BON2 and BON3.

ACA9 is an essential factor for pollen development and fertilization [15]; its possible interactions with copines were also examined. As seen in ACA8 and ACA10, the fused protein of the autoinhibitory domain of ACA9 (1-101aa, Supplementary Materials Figure S1A) with C-terminus of luciferase produced a strong luminescence signal when coexpressed with fused protein of BON1 with N-terminus of luciferase in *N. benthamiana* (Figure S1A). When the fused protein of the autoinhibitory domain of ACA9 (Supplementary Materials Figure S1A) with N-terminus of luciferase coexpressed with the fused protein of BON2 and BON3 with C-terminus of luciferase, a similar strong luminescence signal was observed (Figure 1A). Subsequently, this interaction was further verified by the yeast two-hybrid assay; we found that the C-terminal VWA domain of BON1 (324–578aa), BON2 (328–586aa), BON3 (326–584aa, Supplementary Materials Figure S1A) interact with the segment I of ACA9 (1–101aa, Supplementary Materials Figure S1A,B). 

**Figure 2:**
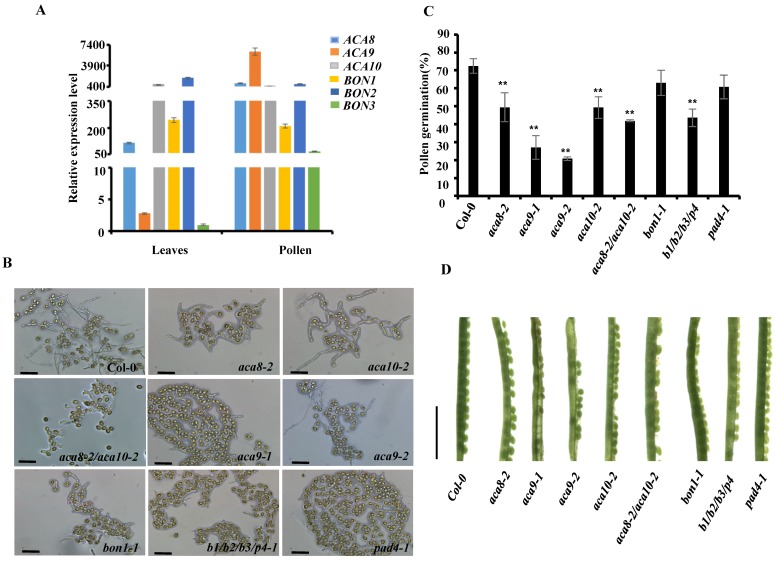
The mutation in *ACAs* and *BONs* affect pollen germination. (**A**). Expression levels of *ACA8/9/10* and *BON1/2/3* in leaves and pollens. (**B**). The phenotype of pollen germination in wild type Col-0 and indicated genotypes (scale bars: 50 μm); (**C**). The pollen germination ratio in various genotypes. The average germination rate was generated from three biological replicates, each replicate with more than 300 pollens. Genotypes: *b1*/*b2*/*b3/p4*=*bon1-1*/*bon2-2*/*bon3-3/pad4-1*. ** represents significant differences compared to WT (*p* < 0.01). (**D**). Seed setting was affected in *aca* and *bon* mutants (scale bar 0.5 cm).

On the basis of the protein–protein interaction, it was speculated that BON1, BON2, and BON3 coordinate with ACA calcium pumps to control pollen development and fertilization. Real-time assay demonstrated that the six genes express both in pollens and leaves with *ACA9* predominantly expressing in pollens (Figure 2A). To test the hypothesis, we characterized the mutants of ACA pumps and copines, focusing on pollen germination. In addition to *aca9* mutant, the *aca8*, *aca10* and *aca8/aca10* mutants exhibited lower pollen germination ratio, suggesting the specific role of different calcium pumps in pollen function (Figure 2B,C). The pollen germination in *bon1* mutant was comparable to wild-type control, suggesting the redundancy between copine genes (Figure 2B,C). Because the triple mutant of *bon1/bon2/bon3* is lethal at seedling stage, we took advantage of *bon1/bon2/bon3/pad4* mutant, in which the autoimmunity is largely rescued by mutation in *PAD4* [22]. Indeed, the pollen germination ratio was largely reduced in *bon1/bon2/bon3/pad4* mutant as compared to wild type (Figure 2B,C), suggesting that all three copines coordinately control pollen germination. Consistent with the defects in pollen germination, the seed production was compromised in *aca8*, *aca10*, *aca8/aca10*, and *bon1/bon2/bon3/pad4* mutants as well as *aca9* mutant (Figure 2C). In summary, two physically interacting family proteins, calcium pumps, and copines coordinately regulated pollen germination and fertility. 

## 3. Materials and Methods 

### 3.1. Plant Materials

The seeds of *bon1-1/bon2-2/bon3-3/pad4-1* were kindly provided by Jian Hua [22]. The *aca8-2* (GK_688H09), *aca9-1* (SALK_045408), *aca9-2* (SALK_108766), and *aca10-2* (GK_044H01) mutants were obtained from the Arabidopsis Stock Centre (http://arabidopsis.org).

### 3.2. Plant Growth Conditions

Arabidopsis seedlings were first grown on half Murashige and Skoog (1/2MS) plates at 21 °C with 1% sucrose for 14 days under 16 h/8 h day/night cycle. The plants were then transferred to soil. The Arabidopsis plants were grown at 21 °C under conditions of 16 h/8 h day/night cycle and relative humidity at 50% to 70%. 

### 3.3. Split-Luc Assay

The opening reading frames of BON1, BON2, and BON3 were amplified using the oligos listed in Table S1. The PCR fragments of BON1, BON2, and BON3 were ligated into the pCAMBIA-NLuc vector digested by *BamHI* and *SalI* using the ClonExpress MultiS One Step Cloning Kit (Vazyme, Nanjing, China) to generate BON1-NLuc, BON2-Nluc, and BON3-Nluc. The PCR fragment of BON2 and BON3 was ligated into the pCAMBIA-CLuc vector, digested by *KpnI* and *SalI*, using the ClonExpress MultiS One Step Cloning Kit (Vazyme, Nanjing, China) to generate BON2-CLuc and BON3-CLuc. The N-terminal parts of ACA8, ACA9 and ACA10 were amplified using the oligos listed in Table S1. The PCR fragments corresponding to the N-terminals of ACA8, ACA9, and ACA10 were inserted into the CLuc vector cleaved by *KpnI* and *SalI* to produce recombinant plasmids of ACA8-I-CLuc, ACA9-I-Cluc, and ACA10-I-CLuc. The PCR fragments corresponding to the N-terminal of ACA9 were inserted into the NLuc vector [24] cleaved by *BamHI* and *SalI* to produce recombinant plasmid of ACA9-I-NLuc. The above recombinant plasmids were transformed into Agrobacterium GV3101.

The agrobacterium with recombinant plasmid was cultured in liquid Luria-Bertani medium with antibiotics (50 mg/L rifampicin and 50 mg/L kanamycin) for about two days. The bacterium was then pelleted by centrifuge and then diluted with Murashige and Skoog medium (10 mM MES and 200 mM acetosyringone) into the concentration of OD_600_ = 0.6–0.8. After 2 h of induction, the agrobacterium with NLuc and CLuc recombinant constructs were mixed in equal volumes and co-transformed into *Nicotiana benthamiana* young leaves. After two days of infiltration under darkness, the leaves were transferred to light for 16 h. The leaves were then sprayed with 1 mM luciferin in 0.01% Triton X100 and kept in dark for 5 min. The luciferase activity was measured using a luminescence imaging system (Sony ICX694, Tanon, Shanghai, China) with a 3 min exposure time.

### 3.4. Pollen Germination Assay

The medium for in vitro pollen germination was modified from a previous report [25] and contained 18% (*w/v*) sucrose, 1.6 mM boric acid, 10 mM CaCl_2_, 1 mM Ca(NO_3_)_2_, 1 mM MgSO_4_, and 0.5% (*w/v*) agarose, pH adjusted to 6.4 with 0.5 M KOH. In vitro pollen germination experiments were conducted at 22 °C under a 100% relative humidity for 6 h [26] and then photographed using a fluorescence microscope (Olypums BX53, Tokyo, Japan). In each replicate, about 300 pollen grains were counted. 

### 3.5. Yeast-Two-Hybrid Assay

The DNA fragments corresponding to the N-terminal segment I of ACA8, ACA9, ACA10 were respectively cloned into the pGADT7 vector (Clontech, Palo Alto, CA, USA). The DNA fragments corresponding to the VWA domain of BON1, BON2, BON3 were respectively cloned into the pGBKT7 vector (Clontech, Palo Alto, CA, USA). To examine the protein–protein interactions, the pGADT7 and pGBKT7 recombinant constructs were co-transformed into the yeast strain AH109. The yeast strains were selected on the SD-Trp-Leu medium and then transferred to the SD-Ade-Trp-Leu-His medium. The colony was photographed after three days growth.

## Supplementary Materials

Supplementary materials can be found at http://www.mdpi.com/1422-0067/19/6/1774/s1.
