# Supplementary figures and images for: Plasma Membrane-Localized Calcium Pumps and Copines Coordinately Regulate Pollen Germination and Fertility in Arabidopsis
Source: Int J Mol Sci. 2018 Jun 15;19(6):1774. doi: 10.3390/ijms19061774 (PMC6032332; doi:10.3390/ijms19061774)

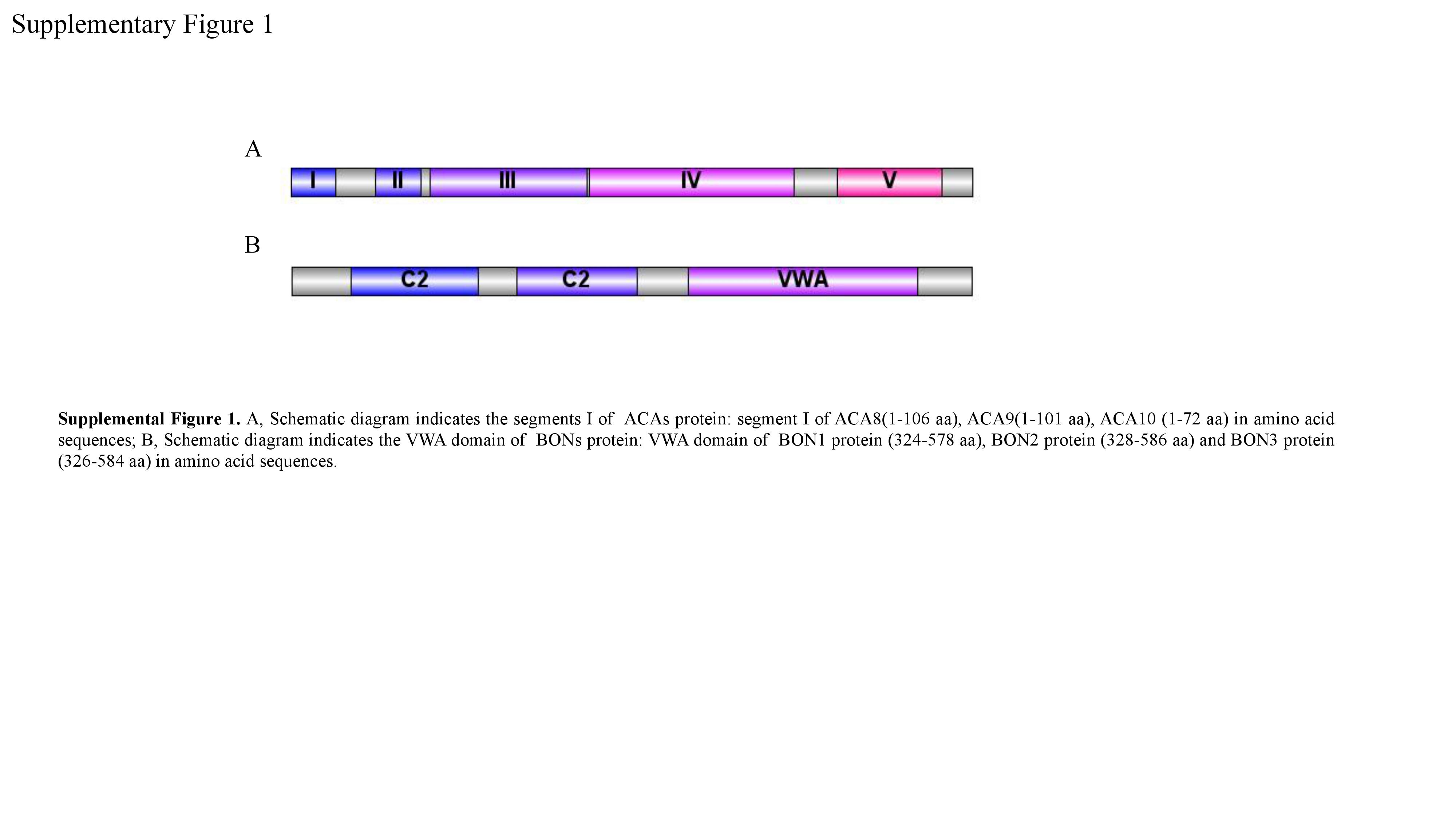

Supplement: Supplementary file 1 [file ijms-19-01774-s001.zip › Figure S1.jpg]

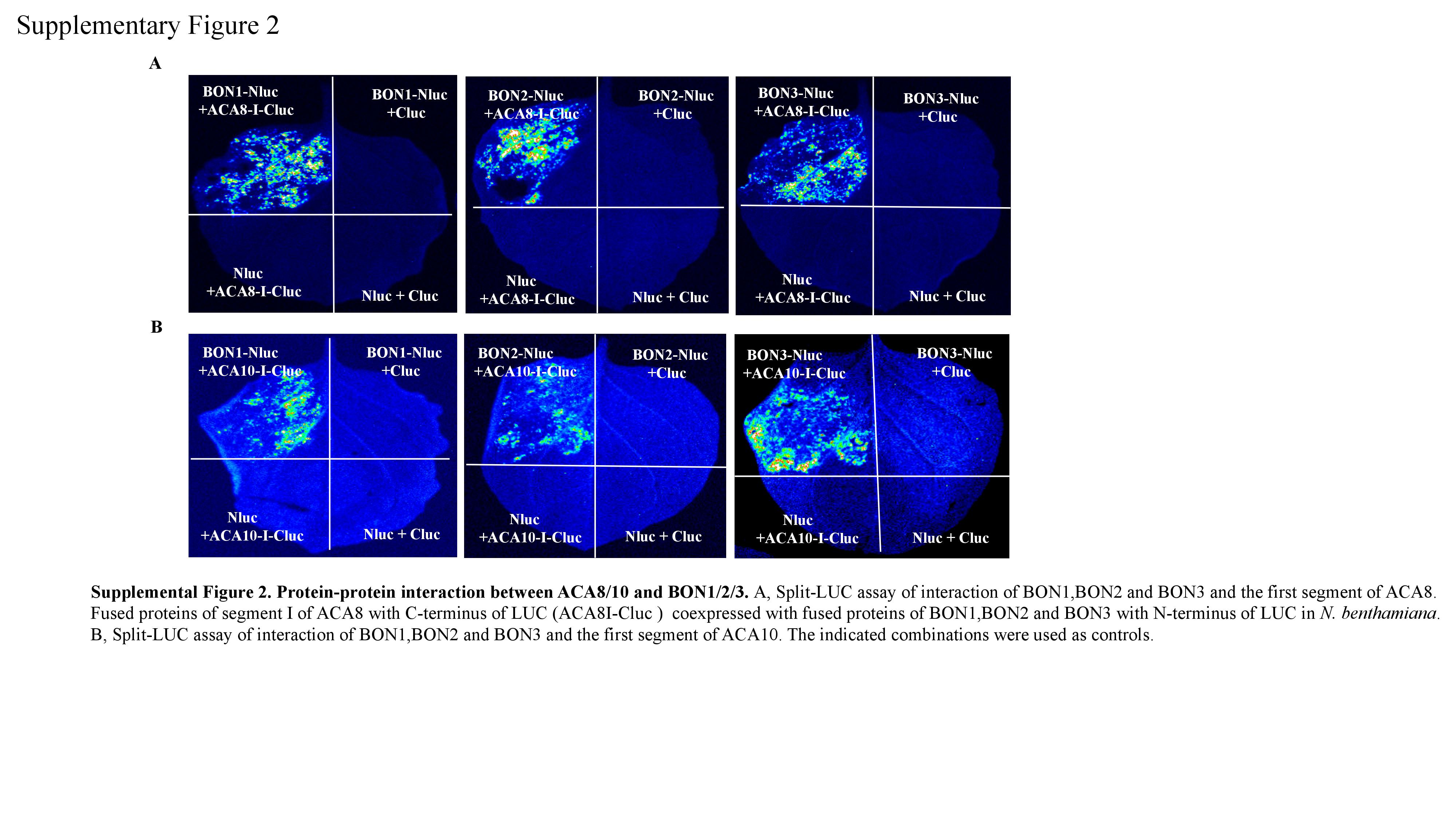

Supplement: Supplementary file 1 [file ijms-19-01774-s001.zip › Figure S2.jpg]

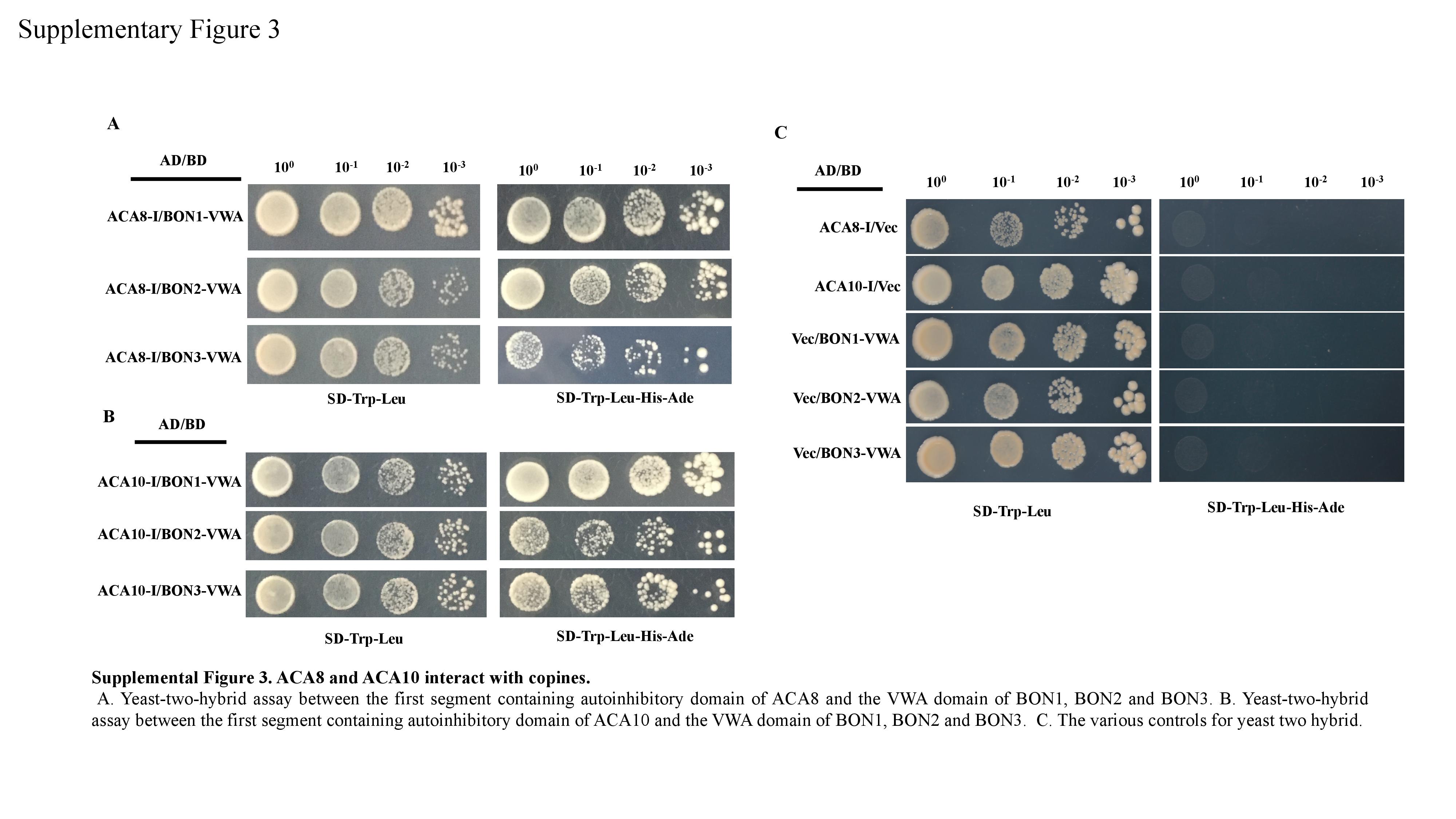

Supplement: Supplementary file 1 [file ijms-19-01774-s001.zip › Figure S3.jpg]
